# Proposal for the applicability of modified Breslow (measured from the basal membrane) as a predictor of survival and sentinel lymph node outcome in patients with cutaneous melanoma^☆^

**DOI:** 10.1016/j.abd.2023.09.002

**Published:** 2024-02-19

**Authors:** Marcel Arakaki Asato, Francisco Alves Moares Neto, Marcelo Padovani de Toledo Moraes, Juliana Polizel Ocanha-Xavier, Luiz Carlos Takita, Mariangela Esther Alencar Marques, José Cândido Caldeira Xavier-Júnior

**Affiliations:** a Faculty of Medicine, Universidade Federal de Mato Grosso do Sul, Campo Grande, MS, Brazil; b Department of Pathology, Faculty of Medicine, Universidade Estadual Paulista, Botucatu, SP, Brazil; c Pathological Anatomy Laboratory, Hospital Amaral Carvalho, Jaú, SP, Brazil; d Faculty of Medicine, Centro Universitário Unisalesiano Auxilium, Araçatuba, SP, Brazil; e Department of Dermatopathology, Instituto de Patologia de Araçatuba, Araçatuba, SP, Brazil

**Keywords:** Melanoma, Neoplasm staging, Pathology, Skin neoplasms

## Abstract

**Background:**

Cutaneous melanoma is a neoplasm with a high mortality rate and risk of metastases to distant organs. The Breslow micrometric measurement is considered the most important factor for evaluating prognosis and management, measured from the granular layer to the deepest portion of the neoplasm. Despite its widespread use, the Breslow thickness measurement has some inaccuracies, such as not considering variations in the thickness of the epidermis in different body locations or when there is ulceration.

**Objective:**

To evaluate the applicability of a modified Breslow measurement, measured from the basal membrane instead of from the granular layer, in an attempt to predict sentinel lymph node examination outcome and survival of patients with melanoma.

**Methods:**

A retrospective and cross-sectional analysis was carried out based on the evaluation of slides stained with hematoxylin & eosin from 275 cases of melanoma that underwent sentinel lymph node biopsy from 2008 to 2021 at a reference center in Brazil.

**Results:**

Analysis of the Cox model to evaluate the impact of the Breslow measurement and the modified Breslow measurement on survival showed that both methods are statistically significant. Logistic regression revealed a significant association between both measurements and the presence of metastasis in sentinel lymph nodes.

**Conclusion:**

Measuring melanoma depth from the basal membrane (modified Breslow measurement) is capable of predicting survival time and sentinel lymph node outcome, as well as the conventional Breslow measurement.

## Introduction

Cutaneous melanoma is a neoplasm that arises from melanocytes and, at an advanced stage, often leads to metastases to distant organs.^1^ The incidence of melanoma has increased in recent decades in light-skinned populations, probably related to recreational behavior and sun exposure. It is believed to arise as a consequence of a complex interaction of environmental and constitutional factors.^2^

The depth of invasion as a prognostic factor was reported by Alexander Breslow in 1970, who demonstrated a correlation between melanoma thickness and risk of recurrence and metastasis.^3^, ^4^ The Breslow micrometric measurement has become the most important factor for prognosis and conduct, is widely used and represents the main factor in staging systems, including that of the American Joint Committee on Cancer.^5^ The Breslow measurement is obtained using a calibrated ocular micrometer to measure, from the most superficial portion of the granular layer to the deepest portion of the tumor.^6^, ^7^ However, the Breslow measurement has some limitations. For instance, when ulceration is present, the Breslow measurement may be underestimated due to the amount of tumor loss that is not taken into account; and, in the absence of the granular layer, as it occurs in the nail region, the measurement can be challenging.^8^ Furthermore, the Breslow measurement does not take into account variations in the thickness of the normal epidermis in different anatomical sites and may show differences even when the invasive component has a similar thickness, as it includes the total thickness of the epidermis.

The depth of invasion is also a prognostic factor for squamous cell carcinoma of the cervix, for instance. In this tumor, however, the depth of tumor invasion is measured from the base of the epithelium, eliminating the influence of the thickness of the epithelium or the presence of ulceration on the final measurement.^9^, ^10^

**Figure 1:**
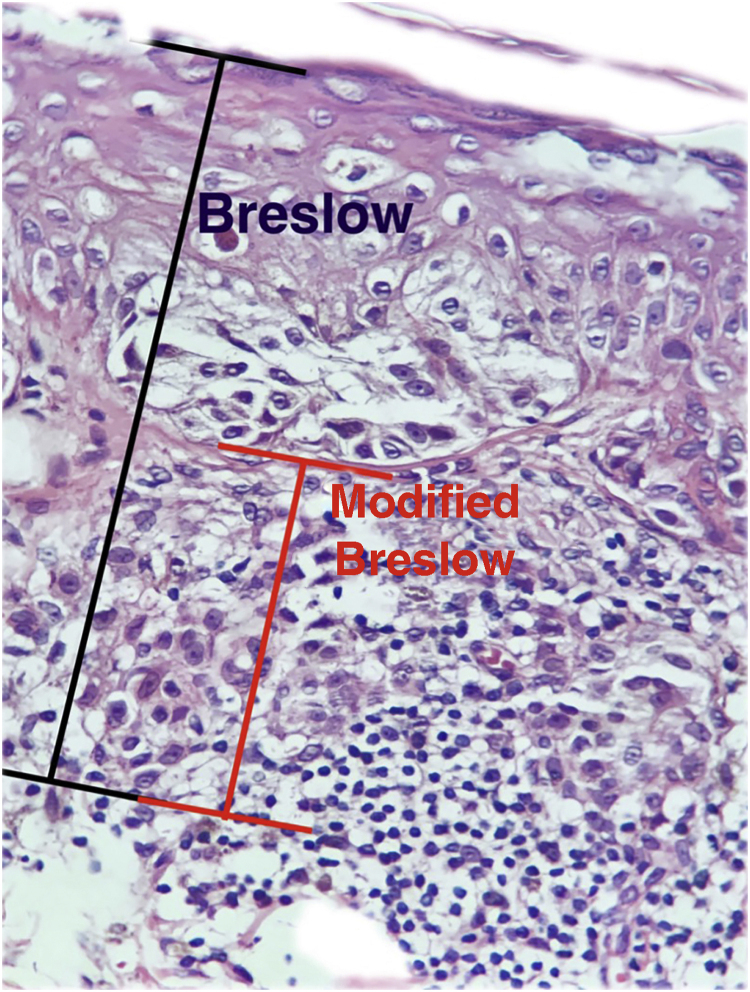
Breslow measurement (in black) compared to the modified Breslow (in red).

The aim of this study was to evaluate the applicability of the modified Breslow measurement, measured from the basal membrane instead of the granular layer, in an attempt to predict the sentinel lymph node outcome and the survival of patients with cutaneous melanoma compared to the classic Breslow measurement (Fig. 1). Additionally, the aim is to evaluate the relationship between sentinel lymph node status (positive or negative for metastases), presence of ulceration, and survival time; and the relationships between anatomical site and histopathological subtype with survival and sentinel lymph node outcome.

## Materials and methods

**Figure 2:**
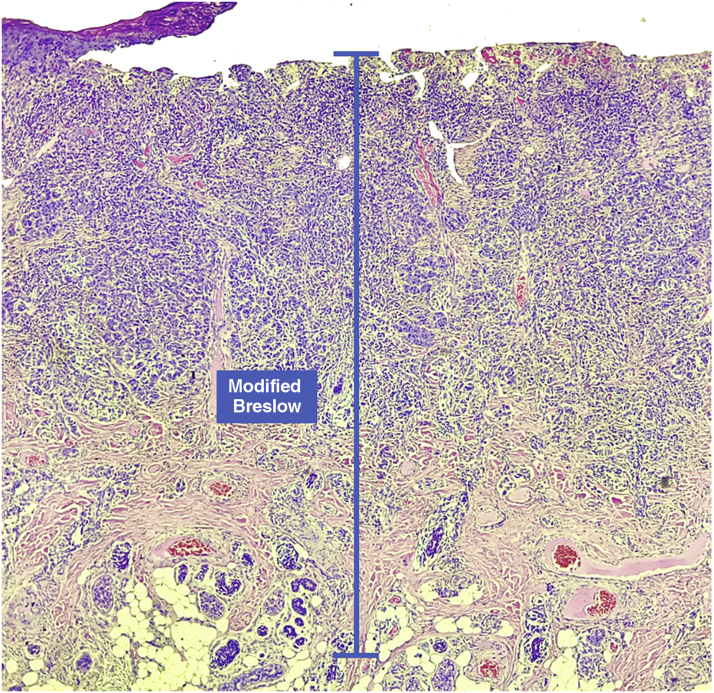
In cases with extensive ulceration, the modified Breslow measurement is taken from the base of the ulcer, as with the conventional Breslow measurement.

This was a retrospective and cross-sectional study that analyzed slides stained with hematoxylin & eosin obtained from formalin-fixed, paraffin-embedded skin samples collected from primary melanoma lesions at Hospital Amaral Carvalho from 2008 to 2021. Clinical and histopathological information (gender, age, location, sentinel lymph node outcome, survival time, and presence or absence of ulceration) were collected from pathology reports stored in the participating institution digital systems. Regarding the topography of the lesion, the cases were divided into: areas not exposed to the sun (anterior chest, posterior chest, abdomen, genital region, and proximal portion of the limbs), areas exposed to the sun (head, neck, and distal portion of the limbs, except the acral region), and acral region (palms of hands, soles of feet and fingers/toes). The microscopic analysis (histopathological type; Breslow measurement; modified Breslow measurement) was performed by two pathologists. The Breslow measurement was measured in the conventional way, from the granular layer to the deepest portion of the neoplasm, while the modified Breslow measurement was measured from the basal membrane to the deepest portion of the tumor, disregarding the “*in situ*” component. In ulcerated cases in which there was an intact epidermis overlying the area of deeper invasion, there was no influence of ulceration in relation to the Breslow measurement. In those cases with extensive ulceration, both measurements were taken from the base of the ulcer (Fig. 2). The exclusion criteria included: *in situ* or thin melanomas (less than 1.0 mm thick), metastases, diagnostic disagreement between pathologists, and those with missing paraffin blocks or with scarce tissue in the paraffin block.

After the data were obtained, a descriptive analysis was initially carried out with the calculation of mean, standard deviation, minimum, maximum, and median values for the quantitative variables and frequencies and percentages for the categorized variables. Considering survival in months, Kaplan-Meier curves were obtained followed by the log-rank test for the variables of interest. In the case of variables with more than two categories, the curves were compared using the Sidak test. Risk factors for survival considering continuous variables were obtained by adjusting the Cox model. For the lymph node as a response variable, a logistic regression model was adjusted considering the Breslow and modified Breslow values as explanatory variables. Associations between categorized variables were assessed using the Chi-Square test. In all tests, the significance level was set at 5% or the corresponding p-value, and all analyses were performed using the SAS program for Windows, v.9.4.

The study was approved by the Research Ethics Committee (REC) of Hospital Amaral Carvalho (CAAE 52618721.0.3001.5434).

## Results

A total of 275 cases of melanoma diagnosed from 2008 to 2021 and which underwent sentinel lymph node biopsy were analyzed. Of these, a total of 141 (51.27%) were men 134 (48.73%) were women and the age median was 63 years (14-87 years). The micrometric Breslow measurement median was 3.8 mm (1.0–27.5 mm), while the modified Breslow measurement median was 3.7 mm (0.5–26.5 mm). The median survival time was two years (0 to 13 years; Table 1).

**Table 1:** Descriptive analysis of the clinicopathological characteristics of cases studied.

Variables	
Sex, n (%)	
Female	134 (48.73)
Male	141 (51.27)
Age (years)	
Median (min.–max.)	63 (14–87)
Breslow measurement (mm)	
Median (min.–max.)	3.8 (1.0–27.5)
Modified Breslow (mm)	
Median (min.–max.)	3.7 (0.5–26.5)
Survival	
In months– Median (min.–max.)	34 (0–158)
In years– Median (min.–max.)	2 (0–13)
Sentinel lymph node, n (%)	
Positive for metastasis	138 (50.18)
Negative for metastasis	137 (49.82)
Anatomical site, n (%)	
Non-exposed areas	100 (36.36)
Exposed areas	84 (30.54)
Acral	78 (28.36)
Not specified	13 (4.72)
Histopathological subtype, n (%)	
Superficial spreading melanoma	101 (36.72)
Nodular melanoma	95 (34.54)
Acral lentiginous melanoma	75 (27.27)
Lentigo maligna melanoma	1 (0.36)
Others	3 (1.09)
Ulceration, n (%)	
Present	144 (59.02)
Absent	100 (40.98)

Of the 275 cases, a total of 138 (50.18%) showed metastasis for the sentinel lymph node. Regarding the anatomical site of the lesion, 100 cases (36.36%) were located in areas not exposed to the sun, 84 cases (30.54%) in areas exposed to the sun, 78 cases (28.36%) in the acral region and 13 (4.72%) in unspecified areas. Regarding the histopathological subtype, 101 cases (36.72%) were superficial spreading melanomas, 95 cases (34.54%) were nodular melanomas, 75 cases (27.27%) were acral melanomas, one case (0. 36%) was lentigo maligna melanoma and three cases (1.09%) were other rare or unclassifiable subtypes. Of the 244 cases with information on ulceration, 144 (59.02%) had ulceration (Table 1).

The results of the Cox model analysis to evaluate the impact of different methods used to measure melanoma thickness (Breslow measurement and modified Breslow measurement) on survival showed that both methods were statistically significant. For the conventional Breslow measurement, a chi-square value of 59.40 was obtained; a p-value < 0.0001; HR of 1.121. For the modified Breslow measurement, a chi-square value of 57.66 was obtained; a p-value < 0.0001; HR of 1.119 (Table 2). The logistic regression disclosed a significant association between both measurements (conventional Breslow measurement and modified Breslow measurement) and the presence of metastasis in the sentinel lymph nodes. The Breslow measurement showed an Odds Ratio (OR) of 1.189 (95%CI 1.111‒1.271; p-value < 0.0001). Likewise, the modified Breslow measurement showed an OR of 1.19 (95%CI 1.112‒1.273; p-value < 0.0001; Table 3).

**Table 2:** Survival analysis using the COX model.

Parameter	DF	Parameter estimate	Standard error	Chi-square	> Chi-square	HR	95% CI of HR	
Breslow	1	0.11437	0.01484	59.4019	<0.0001	1.121	1.089	1.154
Modified Breslow	1	0.11255	0.01482	57.6687	<0.0001	1.119	1.087	1.152

**Table 3:** Logistic regression adjustment for sentinel lymph node outcome for Breslow and modified Breslow.

Variable	OR	95% CI		p-value
Breslow (mm)	1.189	1.111	1.271	<0.0001
Modified Breslow (mm)	1.19	1.112	1.273	<0.0001

**Figure 3:**
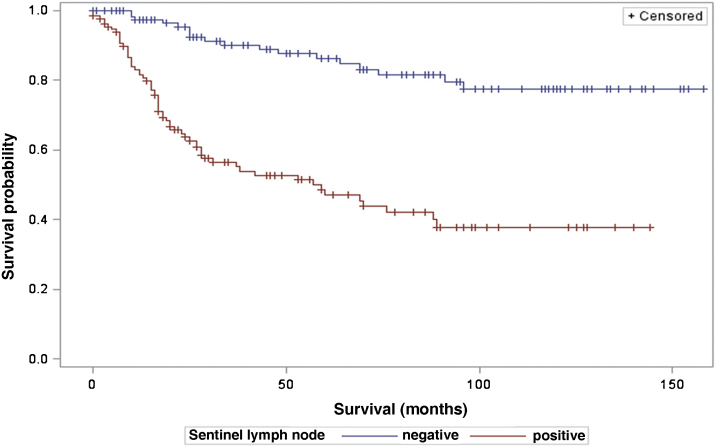
Analysis of survival time in relation to sentinel lymph node status.

**Figure 4:**
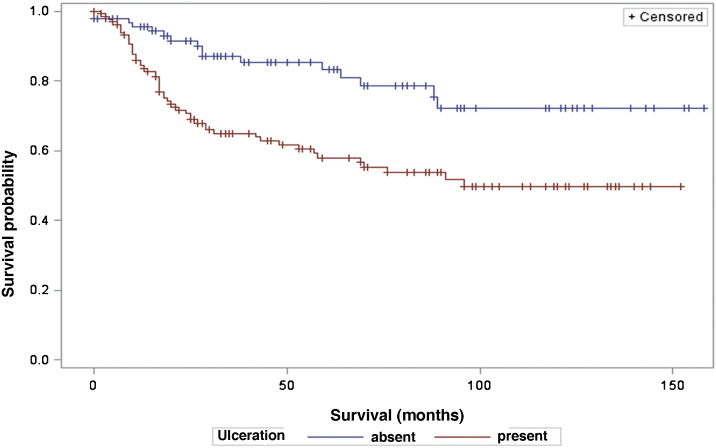
Analysis of survival time in relation to the presence or absence of ulceration.

The analysis of survival time in relation to sentinel lymph node status (positive or negative for metastasis) was performed using the Log-Rank (Chi-Square 39.49; DF 1, p < 0.0001), Wilcoxon (Chi-Square 40.54; DF 1, p < 0.0001) and -2Log(LR; Chi-Square 48.24; DF 1, p < 0.0001; Fig. 3) tests. Likewise, in the survival analysis in relation to the presence of ulceration, the Log-Rank (Chi-Square 11.56; DF 1, p < 0.0007), Wilcoxon (Chi-Square 12.61; DF 1, p < 0.0004) and -2Log (LR; Chi-Square 12.92; DF 1, p < 0.0003; Fig. 4) tests were performed. Moreover, the presence of ulceration showed a significant association with metastases in the sentinel lymph nodes (p = 0.0019).

**Figure 5:**
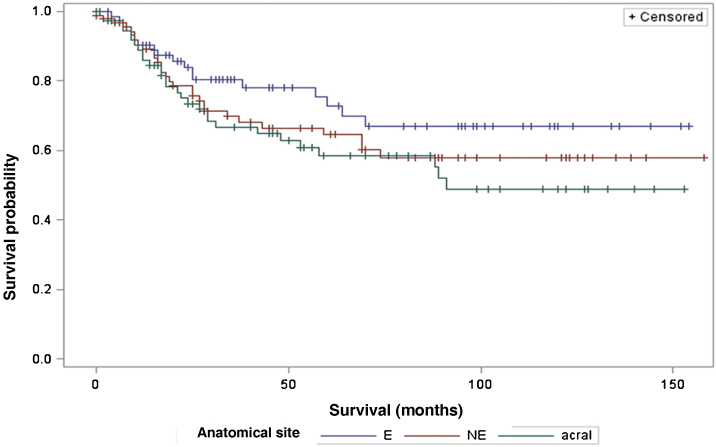
Analysis of survival time in relation to the anatomical site (E, Exposed Areas; NE, Non-exposed Areas).

The survival analysis in relation to the anatomical site did not show any significant results (Fig. 5). The Log-Rank Test obtained a Chi-Square of 2.9495 with DF 2, and a p-value of 0.2288. When adjusting for multiple comparisons using the Sidak Test, none of the pairwise comparisons between anatomical sites reached statistical significance. The comparison between sites exposed to the sun (E) and non-exposed (NE) obtained a p-value of 0.6668 (Chi-Square 1.0447). The comparison between sun-exposed (E) and acral sites resulted in a p-value of 0.2427 (Chi-square 2.9014). Lastly, the comparison between non-exposed (NE) and acral sites resulted in a p-value of 0.9014 (Chi-Square 0.3791). Regarding the anatomical site and its association with sentinel lymph node metastasis, the analysis did not demonstrate any significant differences either (p = 0.1217).

**Figure 6:**
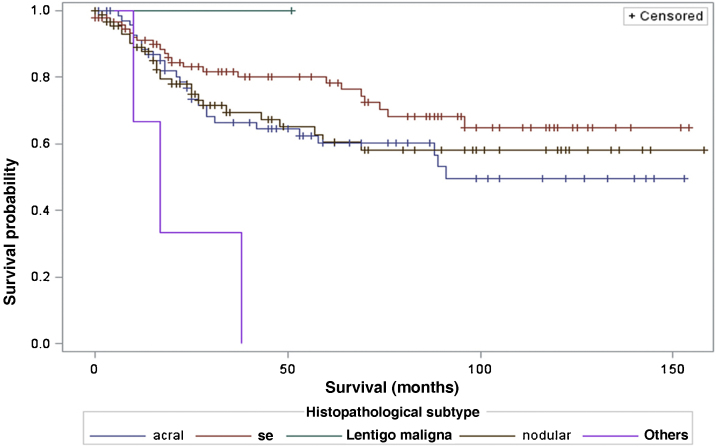
Analysis of survival time in relation to histopathological subtype.

When considering survival in relation to the melanoma histopathological subtypes, the Log-Rank test detected significant differences in the survival rate between different subtypes (Chi-Square 13.11; DF 4 and p-value 0.0107; Fig. 6). Regarding metastases in the sentinel lymph node, there was no statistically significant association between the histopathological subtype (p = 0.0735) and the status of the sentinel lymph node.

## Discussion

The results of this study showed that the modified Breslow measurement (measured from the basal membrane instead of the granular layer) was able to predict survival time and sentinel lymph node outcome, as well as the conventional Breslow measurement.

**Figure 7:**
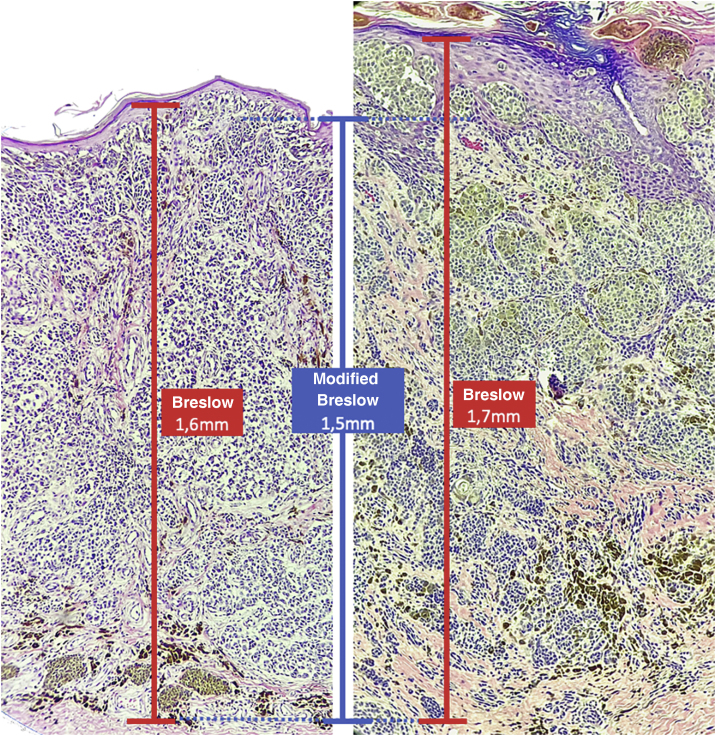
Both cases show an invasive component with similar thickness (1.5 mm, coinciding with the modified Breslow measurement). However, the conventional Breslow measurement is 1.6 mm on the left (thin epidermis) and 1.7 mm on the right (thick epidermis).

The Breslow measure has some limitations. When the lesion is ulcerated, the measurement starts from the base of the ulcer. In these cases, the thickness may be underestimated, as the amount of tumor lost due to ulceration is not taken into account. The current parameters for melanoma staging also do not take into account the thickness of the epidermis when obtaining the Breslow measure. It is known that, for instance, the acral skin has a thicker epidermis, while some areas of the face, such as the skin behind the ear, have a thinner epidermis.^11^, ^12^ In this context, cases of lentigo maligna melanoma, of which the epidermis is generally atrophic, may show a lower Breslow measurement than cases of acral melanomas (since the epidermis of the acral region is thick) even though both show involvement of similar strata in the dermis (Fig. 7). Moreover, the Breslow measurement can only be accurately evaluated in sections perpendicular to the epidermis surface; if there is periadnexal extension of the melanoma and this represents the only focus of invasion, the best methodology for this measurement becomes questionable.^13^, ^14^ The modified Breslow measurement, proposed in the present study, is not affected by variations in epidermis thickness in different anatomical sites, nor by the presence or absence of ulceration (Fig. 7). This approach may show greater reliability, undergoing fewer variations and demonstrating good reproducibility between pathologists.

The results also corroborate that the sentinel lymph node status has a significant effect on the survival of patients with melanoma, and the presence of metastasis in the sentinel lymph node is associated with reduced survival time, in line with other studies such as that of Tejera-Vaquerizo et al., who analyzed 4249 cases of thin melanomas and found that the sentinel lymph node status is the most important prognostic factor for melanoma-specific survival.^15^ Jafari et al., in a study with 1111 patients, showed that those who did not have sentinel lymph node metastases had longer disease-free survival than those who had them. Additionally, in patients with intermediate-thickness melanoma (1.0 to 4.0 mm), better overall survival was found in those with a metastasis-negative sentinel lymph node.^16^ Another study, however, showed that sentinel node status is an independent prognostic factor for disease-free survival, but not for overall survival in a multivariate analysis of 309 cases of melanoma.^17^ Lemos et al., in a study with 43 patients with thick melanoma (> 4 mm), did not demonstrate the statistical significance of sentinel lymph node status in overall survival.^18^

Moreover, ulceration had a significant impact on patient survival, indicating lower survival in those patients with ulceration. These results are in agreement with other studies, such as that by Sarpa et al., with 235 patients, which showed a significant correlation between the extent of ulceration and overall survival as well as sentinel lymph node status.^19^ The study by Hout et al., which showed that both the presence and extent of ulceration are independent predictors of survival.^20^

Regarding the anatomical site of the melanoma, the present study did not demonstrate a significant impact on patient survival, nor any association with the sentinel lymph node outcome. These results are in disagreement with other studies, such as that by Callender et al., with 2500 patients, which demonstrated that the anatomical site is an independent predictor for sentinel lymph node status, as well as survival.^21^ The study by Howard et al., demonstrated that sites with intermittent or chronic sun exposure had better survival compared to sites rarely exposed to the sun.^22^

Regarding the histopathological subtype, there were significant statistical differences in the survival rate between different histopathological subtypes (Kaplan-Meier curve). There was no statistically significant association between histopathological subtype and sentinel lymph node status. The study by Buja et al. showed that the histopathological subtype is an independent risk factor for death, with the nodular subtype being the one with the worst melanoma-specific survival.^23^ Sharouni et al., showed in a study with 48,361 patients that the nodular and acral lentiginous subtypes have worse survival than the superficial spreading and lentigo maligna melanoma subtypes.^24^ The work of Robsahm et al., on the other hand, did not demonstrate histopathological subtype as an independent predictor of melanoma-specific survival.^25^

One weakness of the present study is its retrospective design, the limited number of total cases, the number of ulcerated cases, and the high frequency of advanced-stage cases. Due to a characteristic of the service profile, many cases of acral melanoma and nodular melanoma were also observed, compared to other studies; as well as just one case of lentigo maligna melanoma. Nonetheless, it is the first study to propose the possibility of adapting the way the Breslow measurement is obtained. Thus, the present study demonstrated that the modified Breslow measurement, that is, measured from the basal membrane instead of the granular layer, is capable of predicting prognosis, as well as the conventional Breslow measurement. However, more studies are needed to validate this method.

## Financial support

*Fundação de Amparo à Pesquisa do Estado de São Paulo* (process number 2021/09431-0).

## Authors' contributions

Marcel Arakaki Asato: Data collection; statistical analysis; drafting and editing of the manuscript; collection, analysis and interpretation of data;; critical review of the literature.

Francisco Alves Moares Neto: Critical review of important intellectual content.

Marcelo Padovani de Toledo Moraes: Critical review of important intellectual content.

Juliana Polizel Ocanha-Xavier: Critical review of important intellectual content.

Luiz Carlos Takita: Critical review of important intellectual content.

Mariangela Esther Alencar Marques: Critical review of important intellectual content.

José Cândido Caldeira Xavier-Júnior: Design and planning of the study; effective participation in research orientation; critical review of the literature; critical review of important intellectual content; approval of the final version of the manuscript.

## Conflicts of interest

None declared.
